# Virtual cleaning and unwrapping of non-invasively digitized soiled bamboo scrolls

**DOI:** 10.1038/s41598-019-39447-0

**Published:** 2019-02-19

**Authors:** Daniel Stromer, Vincent Christlein, Xiaolin Huang, Patrick Zippert, Tino Hausotte, Andreas Maier

**Affiliations:** 1 Pattern Recognition Lab, Computer Science, Friedrich-Alexander-Universität Erlangen-Nürnberg, Erlangen, 91058 Germany; 2 Institute of Image Processing and Pattern Recognition, Shanghai Jiao Tong University, Shanghai, 200240 P.R. China; 3 Institute of Manufacturing Metrology, Mechanical Engineering, Friedrich-Alexander-Universität Erlangen-Nürnberg, Erlangen, 91052 Germany

## Abstract

In ancient China, symbols and drawings captured on bamboo and wooden slips were used as main communication media. Those documents are very precious for cultural heritage and research, but due to aging processes, the discovered pieces are sometimes in a poor condition and contaminated by soil. Manual cleaning of excavated slips is a demanding and time-consuming task in which writings can be accidentally deleted. To counter this, we propose a novel approach based on conventional 3-D X-ray computed tomography to digitize such historical documents without before manual cleaning. By applying a virtual cleaning and unwrapping algorithm, the entire scroll surface is remapped into 2-D such that the hidden content becomes readable. We show that the technique also works for heavily soiled scrolls, enabling an investigation of the content by the naked eye without the need for manual labor. This digitization also allows for recovery of potentially erased writings and reconstruction of the original spatial information.

## Introduction

From the Shang dynasty (商朝, 17 century B. C.) to the Jin dynasty (两晋, after 300 A. D.), the main media in China was not paper, writings and drawings were instead captured from top to bottom on bamboo or wooden slips called 简牍^1^. The individual slips, ranging from 20 up to 70, were bound together and rolled up to a scroll forming a chapter of a book. Bamboo was mainly used as writing material because it was abundant everywhere throughout the ancient empire and cheaper than the alternative silk. Depending on the dynasty, symbols were brushed with ink onto the slips or carved into the surface^2^. For brushing, carbon-based inks made of lampblack or soot were produced and formed to inksticks that could then be liquefied again by adding water and rubbing it on inkstone^3^. After the invention of paper and its rapid dissemination across the continent during the Han dynasty (汉朝), bamboo and wooden slips were gradually replaced. Nowadays, such scrolls and slips are a valuable source to increase knowledge of the life of the past.

In the last decades, some major bamboo scroll collections were discovered across China. The Yinqueshan Han Slips (银雀山汉简) were found near Linyi (临沂) in the province of Shandong, where construction workers randomly unearthed the Yinqueshan Han tombs^4^. Around 38 of the discovered slips (1,887 out of total 4,942) were heavily damaged such that a digitization was hardly possible^5^. One of the most important Chinese archaeological discoveries in the last decade was the unearthing of the Liye Qin Slips (里耶秦简) near Liye (里耶), Hunan. More than 37000 pieces with around 200000 recorded characters were found containing a wide range of document types^6^. In terms of numbers, this is not the biggest discovery, but the content of some slips is of very high value. A very famous piece is the world’s oldest example of a multiplication table^7^. The latest significant discovery of bamboo slips was the tomb of Marquis of Haihun (海昏侯). It was discovered 2011 in the Jiangxi province and contained about 5000 slips, bearing for example the ‘Book of medicine’ (医书), the ‘Analects of Confucius’ (论语) or the ‘Classics of filial piety’ (孝经)^8^.

**Figure 1:**
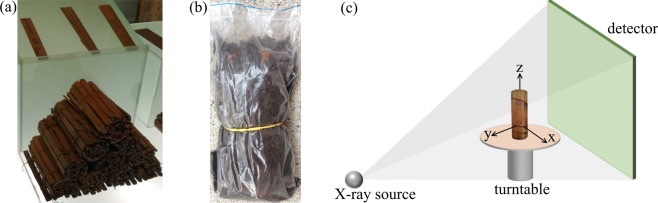
(**a**) Photograph of exemplary bamboo scrolls located at the Changsha Bamboo Slips Museum, Changsha, Hunan. (**b**) Used bamboo scroll covered and filled with potting soil and packed in a plastic bag for the experiments. The carvings were contaminated by pressing the soil onto the surface to create the worst case scenario. (**c**) The 3-D X-ray CT measurement setup: The X-ray source and the flat panel detector are fixed while the object rotates around the *z*-axis. The object is completely covered by the X-rays emitted to the detector such that it can be fully visualized in the reconstructed volume. The scroll is placed upright such that a constant ray length through the object is given aiming to reduce artifacts.

One basic challenge comes with all those discovered historical slips. Due to their age, the slips are in different stages of decay and intact scrolls are found only very rarely^9^. The cleaning process can be very challenging for a conservator because the writing media, as well as the ink and the carvings, can suffer from used chemicals and human imprudence. Figure 1(a) shows a photograph of discovered and cleaned bamboo scrolls located at the Changsha Bamboo Slips Museum (长沙简牍博物馆). The reconstruction of the slips is also very challenging because the strings can be rotten, hydrated, soiled and the slips can be positioned arbitrarily. To reveal the writings of those soiled slips, conservators clean the fragments in a meticulous process. Cleaning and reading unearthed documents by hand is not only a very time-consuming process, furthermore, changing the preserving environment (e. g., brushing off the soils) exposes the slips directly to the atmosphere and they may oxidize rapidly. A preceding non-invasive digitization capable of capturing a digital volume of the entire unprocessed fragile object can help to preserve the valuable content forever without destroying the original structure.

In 2016, Redo-Sanchez *et al*.^10^ used 3-D Terahertz (THz) imaging to read through a stack of paper with promising results. This technique does not expose ionizing radiation to an object, however, it is only well suited for homogeneous materials. As the structure of bamboo scrolls, and especially of surrounding soil or sand, is highly inhomogeneous, the material damping dramatically increases, resulting in a total reflection of the emitted THz waves in the top layers of the object. Furthermore, humidity causes a total reflection of THz waves and as the samples can be highly hydrated, the approach is not suited for prompt digitization in most cases. Given these circumstances, only the surface and a small area underneath can be imaged such that this approach is not suitable for a soiled scroll. However, if only individual soiled slips have to be investigated, this technique is promising for imaging the content underneath the soil layers.

In 2015, Mocella *et al*.^11^ presented a proof-of-concept where they utilized X-ray phase-contrast imaging with Synchrotron radiation for the digitization of a Herculaneum scroll sample. Subsequently, Bukreeva *et al*.^12^ developed an image processing method for virtually unrolling the scroll data. Phase-contrast imaging has also been used for different archaeological purposes, such as scanning cultural heritages^13–15^. Although this technique shows very good results, it is neither widely accessible nor mobile. The discoveries would have to be carried to measurement centers which is costly due to security and insurance reasons.

Previous works on cellulose or collagen-based writing material show that conventional 3-D X-ray computed tomography (CT) systems are well suited for non-invasive imaging when the ink’s X-ray material absorption differs from that of the writing material^16–19^. The increased attenuation is recognizable within the X-ray volume^20^ and applying page segmentation and 2-D mapping algorithms^21^ enable a non-invasive digitization of fragile manuscripts. A disadvantage of this method is the applied X-ray radiation which could accelerate the aging process of cellulose when performing a scan^22,23^. However, the impact of this X-ray radiation is still very limited^24^ and through simulations, the dose can also be kept to a minimum^25^. Immel *et al*. showed experiments on sub-fossils and provide a guideline for X-ray imaging such specimens^26^. Based on an object’s dimension, 3-D X-ray CT reaches resolutions down to 1 μm and even less. Furthermore, this system can easily be carried and mounted at libraries and excavation sites as long as a constant temperature and humidity is given. We showed in an initial proof-of-concept^27^, that utilizing this technique for the digitization of such documents is promising. However, the data processing was performed manually throughout the entire experiment.

For this work, we extended our previous work^27^ in a number of aspects. For the first time, we describe the entire processing pipeline for the digitization of a wrapped up, heavily contaminated bamboo scroll based on a conventional 3-D X-ray CT scan. First, we discuss an appropriate selection of scan parameters based on the material characteristics in detail. Due to corrugated and curved surfaces of the bamboo slips, manual segmentation of the scroll is very time-consuming. We address this issue by proposing a predominantly automatic algorithmic solution. Initially, contamination is erased by a virtual cleaning algorithm. Next, the scroll gets digitally unwrapped and the 3-D slips are finally processed by a texturing step to receive a 2-D representation readable for the naked eye. We show, that our approach is well suited to image and visualize even heavily soiled slip carvings of two exemplary scrolls. If writings or symbols are erased by manual cleaning, the virtually cleaned volume, and the 2-D mapped image can help conservators to identify such spots and recover the information. Furthermore, the volume can be investigated (e. g., by augmented reality) to relocate original slip locations for reconstruction. Based on the scanner and object dimensions, the approach is capable of scanning multiple scrolls at the same time.

## Material Characteristics of Bamboo Scrolls

Obtaining real historical documents is very cost-intensive and hardly possible without conducting initial experiments. Thus, two modern bamboo scrolls bought in Beijing, China, were used. The goal was the investigation of heavily soiled documents. Therefore, the use of replicas is a reasonable way without the need to wait for an excavation or artificially soiling valuable documents. The scrolls are made of bamboo and consist of 32 individual slips, each having a size of 1.2 cm × 15 cm × 0.3 cm (length × width × depth). Rolled up, each scroll has a diameter of about 5.50 cm. The slips have carvings with the depth ranging from 0.10.3 representing either Chinese writings or drawings. The carvings are located on the ‘recto’ surface of the scroll whereas the surface without carvings is called ‘verso’. Scroll A includes a total of 584 Chinese characters denoting traditional Chinese surnames. Scroll B’s 467 characters show the Confucian classic treatise ‘Classic of Filial Piety’ (孝经). The font size ranges from 3 to 6 mm. The ancient scrolls are mostly discovered underground and tend to be contaminated. Therefore, we heavily contaminated the scrolls by potting soil (the used soil also included minerals such as Magnesium or Calcium). We demonstrate that our method still works in the worst case scenario by pressing the soil onto the surface such that the carvings are completely filled. The scrolls were then packed in plastic bags to prevent the scanner from contamination and finally, the bags were completely filled with potting soil. Figure 1(b) shows the described setup of Scroll A. Potting soil, consisting of a mix of cellulose (C_6_H_10_O_5_) and minerals, has a density range of 0.25 to 0.75 g/cm^3^, where the minerals have a rather high density compared to the cellulose particles. For manufacturing scrolls, the bamboo species *Phyllostachys* was a commonly used material due to its hardness grade. Raw bamboo mainly consists of cellulose, hemicellulose, lignin, ash, and other extractives^28,29^. A density distribution profile study of this species revealed a decreasing density from the external to the internal surface, which is caused by the different tissue structures^30^. Although the bulk density of the tested *Phyllostachys* bamboo was about 0.60 g/cm^3^, the outer surface reached densities of more than 1.40 g/cm^3^, whereas the internal surface density dropped to roughly 0.20 g/cm^3^.

## Methods

### Volumetric scanning and reconstruction

In an initial experiment, we showed that industrial 3-D X-ray CT is well suited for non-invasive digitization of the given scroll samples^27^. Although the used scroll sample was heavily contaminated by soil, the carvings could be identified in the output by the naked eye. All scans were performed with a commonly used cone beam geometry micro-CT system (ZEISS METROTOM 1500) consisting of an X-ray source (type: micro-focus tube, focal spot size: >0.007), a flat-panel detector (model: PerkinElmer XRD 1620 xN CS, number of pixels: 2048 × 2048, pixel size: 200 × 200 μm^2^), and a turntable as shown in Fig. 1(c) ^31^. The target material of the built-in X-ray tube was tungsten with the characteristic X-ray spectrum: k*α*_1_ = 59.3 keV, K*α*_2_ = 58 keV, K*β*_1,3,5_ = 67.2 keV, K*β*_2,4_ = 69.1 kev^32^. The turntable rotates around 360 to acquire a set of projection images from which a 3-D volume is reconstructed. round-mode = places,round-precision = 2 The main reason that this concept leads to a readable digitization is an appropriate scan trajectory and parameter selection. The source-detector distance of the setup was 1377.28 mm and the source-object distance was set to 507.44 mm with a half fan angle of 8.4°. The maximum field-of-view of the scanner is 305 × 260 mm^3^ (diameter × height) which is sufficient for the given scroll. When it comes to larger documents, the system provides an image field extension by turntable manipulations increasing the field-of-view to 570 × 550 mm^3^ (diameter × height), however, the scan time will increase for this setup. Also, detector manipulations could lead to an extended field of view^33^.

When scanning objects with 3-D X-ray CT, the reconstructed volume can suffer from artifacts when the X-ray beam length through an object is of high variation^34^. In our case, the scrolls have an approximately constant diameter in *z*-direction, and soil not directly touching the outer layers can be removed carefully. Through an upright scroll positioning (cf. Figure 1(c)), the X-rays penetrate nearly constant material thicknesses reducing the reconstruction artifacts to a minimum.

To calculate the attenuation of an X-ray beam through a material, the Beer-Lambert law is defined as

1 $$I={I}_{0}\cdot {e}^{-\mu d}={I}_{0}\cdot {e}^{-\frac{\mu ^{\prime} }{\rho }\rho d},$$

where *I* denotes the measured beam intensity at the detector, *I*_0_ the incident beam energy of the X-ray source and *d* the thickness of a material. The X-ray mass attenuation coefficient *μ* is calculated by $\mu ^{\prime} \cdot {\rho }^{-1}$ with $\mu ^{\prime}$ denoting the linear attenuation coefficient of material *m* with its mass density *ρ*. The mass attenuation coefficient is a measure of how strong the photons interact with a certain material. In the present case, the object consists of two identical material compositions based on cellulose where only the densities differ. It is assumed that mineral particles are too large to lie in between the carvings as this could not be observed throughout the acquired volumes at any position. Furthermore, the unique structure of bamboo with its decreasing intensities from the recto to the verso surface plays into the X-ray’s hands: when using rather small X-ray energies, the resulting X-ray attenuation of the two components approach a similar or even identical measured attenuation because the photon absorption is rather high for both materials. In contrast, when applying higher X-ray energies, statistically more photons pass through the low-density material than the high-density material. In the given case, where the soil’s and the recto surface’s mean densities differ by approximately a factor of two, higher emitted X-ray energies will lead to improved visibility of the writings as the attenuation of the contamination material in between the very dense carvings vanishes. For our experiments, we selected a tube voltage of 130 kV, a tube current of 1.088 mA, an exposure time of 0.50 s and a projection size of 1,800.

All calculations were performed with the CONRAD framework for cone beam geometries^35^. For the reconstructions, the commonly used Feldkamp, Davis, Kress (FDK) algorithm was applied consisting of cosine weighting, ramp filtering and back projection^36^. For the 3-D case, the approach extends an exact 2-D reconstruction algorithm for fan-beam projections by adapting the cosine weightings. The FDK algorithm performs well for objects that are cylindrical with a small diameter, however, the output may suffer from cone-beam artifacts as it is rather high^37^. These artifacts were not observed throughout our experiments. The resulting maximum voxel size for Scroll A was 103 × 103 × 103 and 163 × 163 × 163 for Scroll B. With the used parameters, the scan and reconstruction time was kept relatively low and can be further reduced by decreasing the projection size or using different trajectories (e. g., short scan trajectory)^31^ or reconstruction algorithms^38^.

### Virtual processing of soiled scrolls

**Figure 2:**
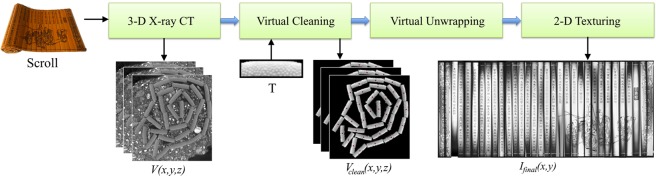
The proposed digitization pipeline: the X-ray CT scan volume is virtually cleaned by providing a manually extracted template image *T*. After virtual unwrapping of the cleaned volume, a 2-D texturing process is performed. The result is a 2-D image of the virtually unwrapped scroll that can be read with the naked eye.

After reconstructing the X-ray volume, the digitized content can be investigated. In case of a soiled relic, handling the acquired data is not intuitive as the individual slips can be arbitrarily located with heavy contaminations along their curved surfaces. One approach to gathering the valuable content from the volume is to manually extract each slip and remove the soil voxel by voxel by hand. The high-resolution volume, however, makes this a very time-consuming issue. In the following section, we propose an image processing pipeline with a minimal amount of user interaction needed resulting in an unwrapped 2-D representation of the scroll’s surface with all writings and drawings readable for the naked eye. The entire digitization pipeline is illustrated in Fig. 2. The unwrapping process can be broken down into three major steps: virtual cleaning, virtual unwrapping, and 2-D texturing.

#### Virtual cleaning

First, the algorithm locates and segments all individual slips of the volume’s scroll resulting in a binary mask where the contamination is removed. This step can be compared with cleaning the scroll in a virtual manner.

**Figure 3:**
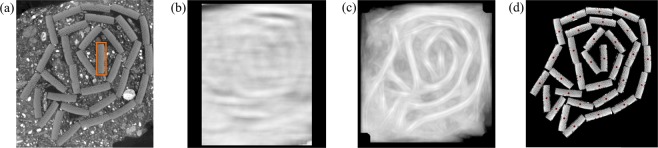
Illustrated virtual cleaning steps for an exemplary volume slice. (**a**) The template *T* is manually extracted by a rectangular selection (orange box) from a central slice. (**b**) NCC Template matching output for a single angle *θ*. (**c**) Output of maximization over all *θ* NCC Template matching results. In comparison to (**b**), the slips’ orientation is clearly visible. (**d**) After smoothing the individual slip center locations (red dots) using RANSAC and PCA, the pixel values within the rectangular template rotated by *θ* is written to the resulting volume yielding a virtually cleaned scroll.

From a central slice of the soiled volume ***V***, a 2-D template *T* has to be extracted manually as shown in Fig. 3(a). The orange rectangular selection denotes the template which is the only necessary manual input for the proposed algorithm. As the appearance and shape of all slips are rather constant in *z*-direction, we apply a normalized cross-correlation template matching (NCC) step along that axis. For a 2-D image *I*, the NCC *R* at image pixel (*x*, *y*) is calculated by evaluating

2 $$R(x,y)=\frac{\sum _{x^{\prime} ,y^{\prime} }(T(x^{\prime} ,y^{\prime} )\cdot I(x+x^{\prime} ,y+y^{\prime} ))}{\sqrt{\sum _{x^{\prime} ,y^{\prime} }T{(x^{\prime} ,y^{\prime} )}^{2}\cdot \sum _{x^{\prime} ,y^{\prime} }I{(x+x^{\prime} ,y+y^{\prime} )}^{2}}},$$

which can be explained by calculating the cross-correlation of the template and the image with subsequent normalization by the sum of the squared differences resulting in a 2-D probability map (0: no match, 1: perfect match). Figure 3(a) shows a single template matched image. As the slips can be arbitrarily rotated within the volume, this step is repeated with the template *T* rotated by various angles *θ* resulting in a probability map *R*_*θ*_. For our experiment, the template was rotated with an angular increment of 0.5° in the range of [0°, 360°]. To get a final probability map *R*_*final*_, the maximum value at each pixel (*x*, *y*) over all *R*_*θ*_’s is extracted:

3 $${R}_{final}(x,y)={{\rm{\max }}}_{{\theta }_{{\rm{\min }}}\le \theta  < {\theta }_{{\rm{\max }}}}({{\rm{R}}}_{\theta }({\rm{x}},{\rm{y}})).$$

**Figure 4:**
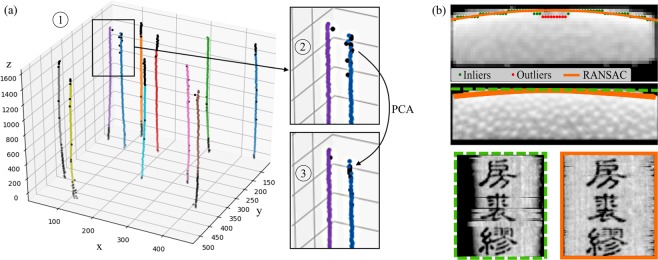
(**a**) All class centroids calculated by the *k*-means algorithm are plotted with different colors (1) and the outliers calculated by RANSAC are marked black. (2) shows a zoom on the box with the black marked outliers which are cropped and (3) the new centroids along the best line fit resulting from the PCA. (**b**) The top image shows the extracted brightest intensities (green and red points) for each column and a RANSAC curve fit (orange line) through the inlier set (green points). The centered and bottom image show a straight line sampling (green) versus curved sampling (orange). Whereas the straight line sampling shows shadows at the horizontal borders, the curve fit also hits the outer parts of the slips. The row-wise artifacts in the bottom left image are caused by slight misalignment due to missing ECC image alignment.

*R*_*final*_ is globally binarized with a threshold (0.9) to extract only maximum response areas. Subsequently, the peak value of a connected area is stored representing the calculated centroid of a slip in the volume. The maximization step’s result *R*_*final*_ of the same slice is shown in Fig. 3(c). It can be seen, that the template rotation process shows a highly improved response map. Furthermore, the highest probabilities denote the center points of the slips and the corresponding stored angle *θ* for each voxel contains the information of the slips’ rotation. To assign class labels Ω_*l*_ to the centroids of all slices, *k*-means clustering^39^ is applied, starting at the central slice $\frac{{z}_{max}}{2}$ and using the previously calculated mean centroids as initialization for the next slice. The number of classes is calculated automatically by assuming the average number of extracted centroids over all slices. Due to noise or X-ray artifacts, the calculated centroids can vary slightly from slice to slice. To receive a smooth output, a Random Sample Consensus (RANSAC)^40^ can help to identify a set of inliers and outliers for all individual *l* slips. Figure 4(a) (1) shows eight randomly selected slip centroids in the volume where colored points denote inliers and black dots outliers. The outliers are rejected and a principal component analysis (PCA) is applied to fit a straight line through the inlier set. This process is shown in the zoomed black boxes of Fig. 4(a) where the outliers calculated by RANSAC were cropped and replaced by the centroids from the PCA resulting in corrected centers, see Fig. 4(a) (3). Finally, with the stored orientation angle *θ* from eq:angle, the template matched region is extracted from ***V*** and written into the final cleaned volume ***V***_*clean*_. This is illustrated in Fig. 3(d), where the red dots denote the calculated peaks from Fig. 3(c) corresponding to the slips’ centroids.

#### Virtual unwrapping

Given the centroids of each slip and their respective rotation from the cleaning step, the scroll can be virtually unwrapped. Therefore, all slips have to be extracted individually from the volume. This can be computed in parallel to the last step of the virtual cleaning. Instead of randomly assigning labels to the individual slips, the center of mass of the volume’s central slice is calculated and the labels are reordered according to the ascending Euclidean distances to the centroids. This approach worked for both our case studies, in cases where the slips are not be connected by strings anymore, this approach might fail. However, the slips can still be manually reordered at the end of the entire processing, as it will not affect the output. The individual slips are then extracted based on the connected centroids through the volume and combined horizontally. The result of this process is an unwrapped and flat three-dimensional scroll volume.

#### 2-D texturing

To provide a readable version of the unwrapped scroll, the individual 3-D slips extracted by the virtual unwrapping step have to be transformed into a two-dimensional representation. As the centroids can still be slightly off-centered and rotated compared to their neighbors (e. g., due to a discrete angular step size), the slip slices are registered on their next neighbor by using the Enhanced Correlation Coefficient (ECC) image alignment technique^41^. The ECC method estimates the warp matrix between two images by maximizing the correlation coefficient between them. This results in a warp matrix which is applied to the neighboring image. Only the upper half of the image containing the recto surface of the document was considered because the vascular bamboo structure could negatively influence the registration results.

The recto and verso layer can be automatically identified by utilizing the material’s density distribution information. An increased density results in higher photon absorption and image intensities and when investigating the data, the recto surface showed increased intensities compared to the verso. Using this information, the recto surface was detected by searching the maximum values along the depth of the ECC aligned image. The top image of Fig. 4(b) illustrates this process where green and red dots denote the extracted maxima corresponding to the recto layer.

It can be observed that the recto layer is slightly curved such that straight sampling would neglect the outermost slip areas. Therefore, we apply RANSAC with a second-degree curve fit onto the set of detected maxima yielding the sampling curve along the surface (Fig. 4(b), orange line). The carvings are correctly detected as outlier set (red dots) and omitted from the curve fit. The central and bottom images of Fig. 4(b) show the comparison of a straight line (dashed green box) versus curved sampling (solid orange box) where the horizontal edges are shaded due to missed recto voxels. Furthermore, the straight-sampled image (bottom left) shows a slip without image alignment resulting in slight geometric differences between neighboring rows introducing row-wise artifacts (especially visible around the image center). The alignment was deactivated to highlight the importance of slip-by-slip registration before sampling. This step concludes the proposed algorithm and results in a 2-D representation of the unwrapped, cleaned scroll *I*_*final*_ where the symbols and drawings are readable by the naked eye.

## Results

For evaluating the proposed digitization pipeline, we first scanned and reconstructed the soiled documents. The scan took about 75 minutes in total for a single scroll. The reconstruction was performed in parallel to the scan such that the final reconstructed volume was available seconds after the last projection was generated. We initially cropped the volume to a cuboid including all the relevant information to reduce the large amount of data. Next, we determined the template image from a central slice of Scroll A and applied the proposed algorithm as illustrated in Fig. 2. Throughout the entire pipeline, the algorithms were implemented with CPU multi-threading. Processing the entire volume took approximately 280 minutes (template matching: 278 min, *k*-means: 28 s, RANSAC: 17 s, PCA: 2, 2-D texturing: 2 min). The method’s bottleneck is the template matching process of the volume ${\boldsymbol{V}}\in {{\mathbb{R}}}^{X\times Y\times Z}$ with a complexity of approximately *O*(*θXZN*^2^) where *N* denotes the template’s dimension (*N* = 36 × 118, *θ* = 720, *X* × *Y* × *Z* = 548 × 586 × 1544). In order to reduce the runtime to a minimum, we increased the angular increment to 1 and downsized the template as well as the *xy*-slice of the volume by a factor of 4 for the template matching process. With these adaptations, the runtime decreased to a total of around seven minutes, which is acceptable and does not influence the output significantly. A GPU-based implementation could further reduce the runtime.

**Figure 5:**
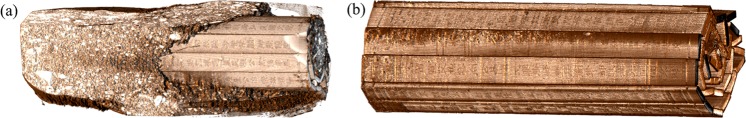
(**a**) Reconstructed and rendered soiled Scroll A volume. The scroll is mostly covered and filled with soil, however, due to gravity, some slips and their carvings are partly visible. (**b**) The rendered volume after virtual cleaning is shown. Nearly all contaminations were removed by the proposed algorithm such that the individual slips are visible.

The rendered raw volume of the 3-D X-ray CT scan can be seen in Fig. 5(a) where due to gravity, the scroll was partly uncovered. The resulting virtual cleaned volume is shown in Fig. 5(b). Here, the contaminations are mostly removed and only small areas on the scroll’s surface are still covered. However, this will not affect the final output and investigations on the slips’ orientation can be applied.

**Figure 6:**
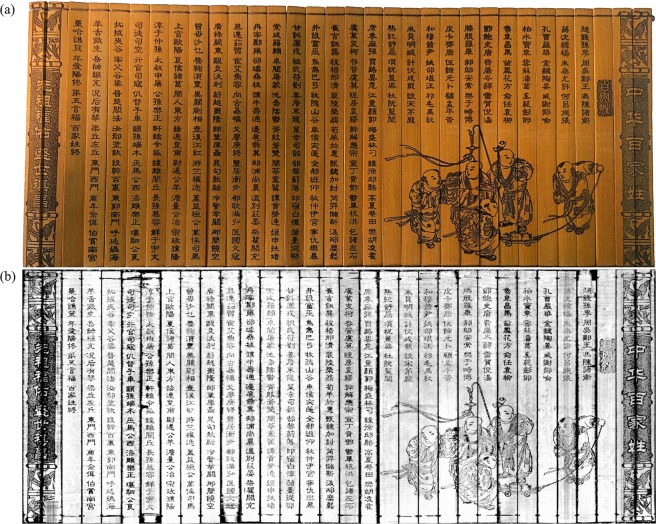
(**a**) A photograph of the manually unwrapped original Scroll A. The sample was rolled up, soiled and digitized by the proposed pipeline. (**b**) The final 2-D output of the reconstructed and processed soiled Scroll A. Although the scroll was heavily contaminated with potting soil, most characters and drawings are clearly visible.

**Figure 7:**
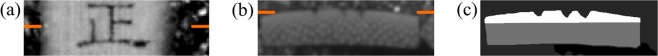
(**a**) Randomly selected carving slice of an extracted slip. At the position of the orange lines, a depth view was extracted shown in (**b**). The depth view shows the slip surrounded by air and soil. The segmentation map is shown in (**c**). Recto layer (white), verso layer (light gray), soil (dark gray) and air (black) were manually segmented. An analysis of the mean intensities of the areas reveals that the recto layer’s attenuation differs by 29% from the soil’s one.

Next, the unwrapping and 2-D texturing steps were performed for receiving a readable version of Scrolls A content. Figure 6(a) shows a photograph of the unwrapped Scroll A before soiling and wrapping. The virtually cleaned, unwrapped and 2-D textured image *I*_*final*_ of the recto surface is shown in Fig. 6(b). As can be seen, most of the characters and drawings are readable without application of any post-processing steps. The curved sampling correctly remaps the surface voxels to the 2-D image and all slips appear smooth and in the correct order. To highlight the X-ray attenuation difference of the same material with varying densities, an exemplary measurement of a single slice was made. Figure 7(a) shows the randomly selected exemplary carving ‘’ (translation: ‘positive’). The depth section was extracted at the position of the orange line and shown in Fig. 7(b) (here, the orange lines correspond to the illustrated layer of Fig. 7(a)). To compare the photon absorption of the different material densities, four layers were investigated by calculating their mean intensity within the image. These manually segmented layers are shown in Fig. 7(c). The black area corresponds to air with a mean intensity of 3.48. The dark gray area denotes soil with a mean of 4.47. Brighter spots within this area are minerals with higher attenuation than cellulose. The slip was divided into two areas – the verso and recto layer. The recto surface has a mean intensity of 5.49 whereas the verso layer reaches 4.95. These measurements were performed on a random basis for several slices with about the same results. The analysis of the intensities validates the observation that the recto surface has a higher attenuation than the verso layers such that the recto can be identified automatically. When normalizing the mean values according to air, the mean soil X-ray attenuation is 29% higher, whereas the mean recto surface density is even 58% higher. This increase of attenuation leads to the contrast difference between bamboo and carving uncovering the writings and drawings.

**Figure 8:**
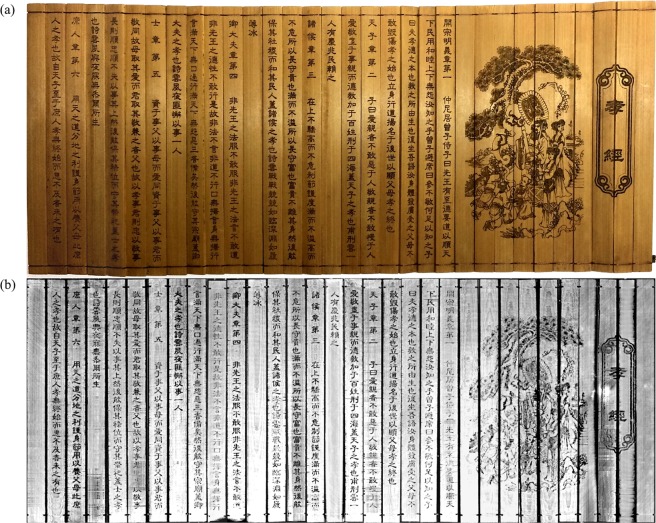
(**a**) A photograph of the manually unwrapped original Scroll B. Also this sample was rolled up, soiled and digitized by the proposed pipeline. (**b**) The final 2-D output of the reconstructed and processed soiled Scroll B. Although the scroll was heavily contaminated by potting soil, most characters and drawings are clearly visible.

Finally, we tested the algorithm for Scroll B where the photograph before contamination can be seen in Fig. 8(a). Instead of extracting a template again, the one from Scroll A was used as input, since the scrolls differ only slightly. The results after applying the digitization pipeline are shown in Fig. 8(a). Once more, all writings are readable, however, the drawings appear blurry. This is due to the lower resolution of this scan where the object was placed closer to the X-ray source reducing the magnification. The slips were ordered correctly and were not flipped within the final image.

The results show that the non-invasive digitization of excavated, heavily soiled bamboo slips by 3-D X-ray CT is highly promising when the writings and drawings are carved into the bamboo. By using the proposed unwrapping algorithm, a minimal amount of manual effort is needed to receive a readable, digital version of the scrolls content.

## Discussion

In this work, we presented a complete pipeline for a non-invasive digitization of soiled bamboo scrolls composed of individual bamboo slips. Our method may enable libraries and conservators to preserve the structure and writings or drawings of historical bamboo and wooden scrolls. The approach is based on a conventional 3-D X-ray CT scan followed by a fast unwrapping algorithm. Such X-ray systems are widely accessible and more mobile compared to phase-contrast systems. The scan as well as the proposed algorithm were tuned to address the unique material characteristics of bamboo. With the user only providing a template image of a single slip, the algorithm’s output is a 2-D image of the scroll’s recto layer containing the carvings. We could show that even when the documents were heavily soiled by material of similar chemical structure, their hidden content could be made readable for the naked eye. With the proposed technique, there is no need to manually clean the scroll before scanning. If such a scan is performed before the scroll is uncovered by a conservator, writings possibly erased by the cleaning process are stored and could be recovered instead of being lost forever.

Until now, only carved writings were investigated. Ink brushed onto the surface without being carved into the surface can only be made readable digitally, if the ink is composed of a material with different X-ray attenuation than the document media and its surrounding. Also the density difference of cellulose and soil is rather small. To improve the contrast between document, writings, and soil, phase-contrast and X-ray dark-field imaging can be used instead of conventional X-ray CT. Both methods are able to reveal unique information about structural variations at sub-micron scale^42–46^.

The digital volume generated by 3-D X-ray CT can be very helpful for conservators. Usually, the slips are arbitrarily located in the soil due to rotten strings and material decay and only very few cases exist where the entire scroll was found intact. According to works of E. Giele^47,48^, determining the original composition of the slips is a very challenging but highly important task for researchers, who have to mark the excavated slips, draw a location map, and reconstruct everything within their laboratory. T. Staack^9^ shows an exemplary drawing. The work also mentions brushed or knife-cut lines on the verso of slips (‘verso-lines’), which can indicate the original position of a slip. Furthermore, mirrored writings on the verso were present, imprinted by a touching slip’s recto. When using the proposed non-invasive CT technique, those imprints could be automatically detected by applying image transformations and registration approaches. In addition, all information is stored in a digital manner and methods such as augmented reality or 3-D printing of the virtually cleaned volume can be used to further investigate the original spatial information. The technique can be extended for all kinds of woodcuts, archaeological findings or forensics when the material composition differs from the contamination material.

The generated data can be a basis for future algorithms in the field of document processing. Therefore, the acquired scan data and the code will be made publicly available to enable future research. We will also create a publicly available database with more scan data, where the data from this work could serve as ground truth segmentation for machine-learning algorithms. All generated data and code from this work will be made available at https://www5.cs.fau.de/research/data/bamboo-scroll-dataset/.

The proposed work provides a basis for non-invasive digitization of ancient bamboo and wooden scrolls where the greatest challenge lies in the high variability of the excavated documents and the surrounding soil. For future work, we plan to investigate real historical slips or scrolls. The success of imaging those excavated scrolls with the proposed method depends on their state of decay. Fengel *et al*.^49^ state that fossilized timbers showed decreased densities and Hamid *et al*.^50^ showed that some bamboo species are more resistant to fungi than others. Hence, the density of bamboo slips is highly depended on the used bamboo species and the surrounding soil. It follows that the attenuation values of both materials approach similar levels. The proposed slip detection method (virtual cleaning) is mainly based on the appearance of the slips. When it comes to differing appearances of slips in the volume due to deterioration, the proposed algorithm will no longer detect the writing media. In this case, machine-learning algorithms could help to virtually clean the volume by turning the process around: the conservators could scan an amount of surrounding soil from nearby the excavation location. Based on this data, an algorithm could be trained to learn the soil’s appearance and afterward segment the scroll in the scroll-included volume.
